# Constipation and Laxative Use among Nursing Home Patients: Prevalence and Associations Derived from the Residents Assessment Instrument for Long-Term Care Facilities (interRAI LTCF)

**DOI:** 10.1155/2016/1215746

**Published:** 2016-01-17

**Authors:** Lene Elisabeth Blekken, Sigrid Nakrem, Anne Guttormsen Vinsnes, Christine Norton, Siv Mørkved, Øyvind Salvesen, Kari Hanne Gjeilo

**Affiliations:** ^1^Faculty of Health and Social Science, Department of Nursing Science, University College of Sør-Trøndelag, Postboks 2320, 7004 Trondheim, Norway; ^2^Faculty of Medicine, Department of Public Health and General Practice, Norwegian University of Science and Technology (NTNU), Postboks 8905, MTFS, 7491 Trondheim, Norway; ^3^King's College London, Florence Nightingale School of Nursing and Midwifery, 57 Waterloo Road, London SE1 8WA, UK; ^4^St. Olavs Hospital, Trondheim University Hospital, Clinical Service, Postboks 3250 Sluppen, 7006 Trondheim, Norway; ^5^St. Olavs Hospital, Trondheim University Hospital, Department of Cardiology and Department of Cardiothoracic Surgery, Postboks 3250 Sluppen, 7006 Trondheim, Norway; ^6^Faculty of Medicine, Department of Circulation and Medical Imaging, Norwegian University of Science and Technology (NTNU), Postboks 8905, 7491 Trondheim, Norway

## Abstract

*Introduction*. Constipation is a common, bothersome, and potentially dangerous condition among nursing home (NH) patients. Between 50 and 74% of NH patients use laxatives.* Objective*. To study prevalence and associations of laxative use and constipation using the comprehensive Norwegian version of the Resident Assessment Instrument for Long-Term Care Facilities.* Methods.* Cross-sectional study. Patients from 20 NH units were included. Logistic regression was used to analyze the results. Data collected in NHs might be clustered. Consequently, the multivariable models were tested against a mixed effects regression model to investigate variance both on the level of patients and on the level of NH units.* Results.* In all, 261 patients were included. The prevalence of constipation was 23.4%, and 67.1% used laxatives regularly. Balance problems, urinary incontinence, hypothyroidism, and Parkinson's disease were associated with constipation. Reduced ability to communicate and number of drugs were associated with laxative use. Antidementia-drugs and being involved in activities 1/3 to 2/3 of daytime were protective factors for laxative use. Mixed effects analyses identified variance on the level of NH units as nonsignificant.* Conclusion.* Constipation and laxative use are common. Variance is mainly explained by different patient characteristics/health deficiencies. Hence, patients might benefit from individualized care to compensate for deficiencies.

## 1. Introduction

The management of constipation among patients in nursing homes (NHs) is challenging for both patients and health care staff [1]. Constipation is not a well-defined disease, but a general term describing the difficulties a person experiences with their bowel movements [2]; thus epidemiological studies show great disparity in the reporting of prevalence. The prevalence of constipation increases with age, with the largest increase in prevalence after the age of 70 years [3, 4]. Women are 2-3 times more likely to have constipation than men [3, 4]. Between 17 and 40% of the community-dwelling older adults [5–7] and between 10 and 72% [8–11] of NH patients experience constipation.

Constipation can be classified as primary (idiopathic or functional) or secondary (iatrogenic or because of organic disease), the latter being more common in older people [12]. Diseases associated with constipation are endocrine or metabolic disorders; gastrointestinal disorders; neurological disorders; and psychological comorbidities [4]. Other contributory factors to the higher prevalence of constipation among older people include poor dietary fibre, fluid and calorie intake, immobility, weak abdominal and pelvic muscles, and cognitive impairment and medication side effects [12]. Among NH patients constipation is associated with impaired health-related quality of life [13–16], physical aggression [17], and psychological distress [16]. Chronic constipation can lead to faecal impaction [4, 6], and in severe cases, faecal impaction can cause stercolar ulcerations, intestinal obstruction, or bowel perforation [1]. Other complications of constipation are related to excessive straining that can contribute to haemorrhoids, anal fissures and rectal prolapse. Excessive straining can affect the cerebral and coronary circulation with resultant syncope or cardiac ischemia [12]. Some age-related changes in anorectal physiology have been described [2]. However, constipation should not be regarded as a physiological consequence of normal aging, since most healthy older people have normal bowel function [2]. Nurses working in NHs report constipation as hard to manage due to busy working days with many tasks, so that good bowel routines have low priority [18]. Further, staff discontinuity and a high proportion of unskilled nursing aides among the staff hinder good management of the patients' bowels [18, 19].

In addition to conservative interventions such as dietary fibre, physical activity, and fluids, laxatives are the cornerstone in the treatment of constipation. Between 50 and 74% of NH patients are reported to use laxatives regularly [4, 8]. All groups of laxatives are superior to placebo [20]. However, in contrast to the overall good results in clinical trials, patients' satisfaction with everyday use of laxatives is rather low [21]. Laxatives may serve as a marker for constipation because they are rarely used for other indications. Indeed, several NH studies have used laxatives as a proxy marker for constipation [8, 22–24]. In addition, constipation is a significant driver of health care costs including laxative use and time resources for health care personnel dealing with the problem in hospitals and NHs [25, 26]. In Norway, with a population of approximately 5.2 million, 18.9 million € was spent on laxatives in 2014 [27]. Constipation is a multifactorial condition with huge variability in reported prevalence in the NH population. There is therefore a need to investigate the condition with validated instruments. The Resident Assessment Instrument for Long-Term Care Facilities (interRAI LTCF) [28] is a standardized, validated and comprehensive tool to assess patients' health condition in the long-term care setting, which allow for international comparability. In addition, it is poorly understood whether clustering of observations in NHs affects the results [29, 30], and whether variability in prevalence found is due to differences between patients or differences between NH units [31].

The aim of this study was to study prevalence and associations of constipation and laxative use among NH patients using the Norwegian version of the interRAI LTCF [28]. A secondary aim was to investigate the effect of clustering of observations and whether living in different NH units had an impact on the prevalence of constipation and laxative use by analysing data using mixed effects models.

## 2. Materials and Methods

### 2.1. Design

A cross-sectional design was employed. The study was performed in NHs in one urban municipality in Norway, during September and October 2014. Data were collected at baseline in an ongoing cluster-randomized controlled trial investigating the effect of an educational program for care staff about faecal incontinence in NH patients [32]. Sample-size calculations for the trial are reported elsewhere [32]. The trial is registered in the clinical trial registry (NCT02183740).

### 2.2. Setting

Most Norwegian NHs are owned and run by the municipalities, are oftentimes managed by Registered Nurses (RNs), and have an agreement with a general practitioner (GP) who visits the NH once a week. There are no legal requirements for staff-to-patient ratios or specifications for qualifications required for care workers [33]. However, NHs have RNs on duty 24 hours a day, and according to Statistics Norway the staff comprises on average 31% RNs, 45% licenced practical nurses who are care staff with high school education, and 24% healthcare aides with no formal health care education [34]. In Norway, a majority of NH patients are above 67 years and have complex health problems, significant deficiencies in functioning related to activities of daily living (ADL), and about 80% have cognitive impairment [35].

### 2.3. Patients

Patients were recruited from NHs. Out of a total of 27 NHs available in the municipality, 20 NH units from 10 different NHs were recruited. All NHs had 24 hour long-term residency, comparable staff-to-patient ratios on the day shift and similar GP coverage. Specialized NH units or units with enhanced staff-to-patient ratios were excluded. All long-term care patients with a stay of four weeks or more were eligible for inclusion.

### 2.4. Variables

The interRAI LTCF is a standardized, validated, and comprehensive tool to assess patients' health status in the long-term care setting [28, 36–38]. In this study interRAI LTCF sections C to O were included, and the following variables were used.

*Constipation* was measured by interRAI LTCF, section J: Constipation, defined as no bowel movements for three days or problems with hard stools. Based on this definition, the RNs coded 0 for not constipated, 1 for problems with constipation, but no symptoms the last three days, 2 for symptoms of constipation present 1 of the last 3 days, 3 for symptoms of constipation present for 2 of the last 3 days, and 4 for symptoms of constipation present daily for the last 3 days. For this study, all patients with the scores 1 to 4 were defined as constipated.

*Laxative use* prescribed as regularly used in the patient record and recorded in interRAI, section N: Medications, and grouped according to the Anatomical-Therapeutic-Chemical Classification System (ATC) [39] (see (xi) below).

Informed by other studies [4, 6] the following variables from interRAI were used to investigate possible associations:

- Patients' cognitive status was measured by the Cognitive Performance Scale (CPS) [40]. Scores range from 0 to 6, where 0 represents being cognitively intact. To define presence of cognitive impairment, the usual cutoff of 2 points or more was used [41].
- Patients' functional status was measured by the Activities of Daily Living long form scale (ADLlf) [42]. The items in this study differ from the original scale due to differences in ADL items in the Norwegian version of the interRAI LTCF. After communicating with a Norwegian member of the interRAI organization, the following 7 items were included: personal hygiene, dressing upper body, dressing lower body, locomotion, toilet use, eating, and bed mobility. Scores range from 0 to 28, where 0 indicates no functional difficulty.
- The Depression Rating Scale (DRS) [43] was used to measure depression symptoms. Scores range from 0 to 14, where 0 indicates no depression symptoms.
- Patients' instability in health/frailty was measured by the Changes in Health, End-Stage Disease, Signs, and Symptoms Scale (CHESS) [44]. Scores range from 0 to 5, where 0 indicates stability in health.
- The Aggressive Behavior Scale (ABS) [45] was used to measure aggressive behavior. Scores range from 0 to 12, where 0 indicates no aggressive behavior.
- The Revised Index for Social Engagement (RISE) was used to measure the degree of involvement in positive social activities [46]. Scores range from 0 to 6, where 0 indicates no involvement in positive activities. Compared to the other scales derived from interRAI LTCF, this is the only scale where low score is worst rather than best.
- The communication scale (COMM) was constructed by summing the scores for the variables “expressive communication skills” and “receptive communication skills,” each with a score range of 0 to 4. This resulted in a score range for COMM from 0 to 8, where 0 indicates no communication problems [47].
- Four variables measuring Balance in section J3 were used to construct a scale to measure balance: the four variables were: “Has difficulties or is unable to move to the standing position without help”, “Has difficulties or is unable to turn to the opposite direction when standing”, “Dizziness”, and “Walking instability”. The individual variables were dichotomized and the scores then summed giving a score range from 0 to 4, where 0 indicates no balance problems.
- The following individual interRAI LTCF variables were used: “Faecal incontinence”, “Urinary incontinence”, “Pressure ulcers”, “Maximum walking distance”, “Locomotion”, “Activity level”, “Fatigue”, “Body mass index”, “ Dehydration”, Type of food (Regular or soft/liquid diet).
- The patients' medical condition was measured by section I of interRAI LTCF: “Alzheimer's disease”, “Dementia other than Alzheimer's disease”, “Hemiplegia”, “Multiple sclerosis”, “Paraplegia”, “Parkinson's disease”, “Quadriplegia”, “Cerebrovascular accident (stroke)”, “Cardiovascular disease”, “Congestive heart failure”, “Chronic obstructive lung disease”, “Anxiety disorder”, “Bipolar disease”, “Depression”, “Schizophrenia”, “Pneumonia”, “Urinary tract infection”, “Cancer”, “Diabetes mellitus”, “Hypothyroidism”. Comorbidity is measured by summing the above diagnoses giving one point per diagnosis.
- Medications were measured by section N of interRAI LTCF and grouped according to the ATC-System, primarily on level four since drugs at this level often have common adverse drug reactions [48]: “opiates (N02A)”, “antiepileptics (N03A)”, “antipsychotics (N05A)”, “anxiolytics (N05A)”, “hypnotics and sedatives (N05C)”, “diuretics (C03)”, “antidepressants (N06A)”, “anti-dementia drugs (N06D)” “iron supplements (B03A)”, “calcium supplements (A12A)” “antidiarrheal agents (A07D)” and “laxatives (A06A)”. Groups of laxatives were defined at ATC-level 5: softening laxatives (A06A A), stimulant laxatives (A06A B), bulk laxatives (A06A C), osmotic laxatives (A06A D), and enemas (A06A G).

In addition, the questionnaire included a section where RNs were offered a list of interventions relevant for constipation and asked to identify what is done for each individual patient. This list included questions about administration of laxatives (tablets, oral liquid, and suppositories) and enemas.

### 2.5. Data Collection

The project coordinator and a research assistant gave information and training to RNs (2-3 hours per NH) on completion of all the measures listed above. RNs were trained to use the interRAI LTCF standardized coding guidelines provided in the instrument's training manual. RNs used clinical judgment together with information from the electronic patient record, coworkers, and the patients when filling in the questionnaire.

### 2.6. Statistics

Statistical methods included estimating prevalence in percentages and other descriptive statistics. InterRAI LTCF offers a large number of variables. Univariable logistic regression analysis was conducted on the variables identified under* data collection*. We used perceived clinical significance, log likelihood, McFadden's *R* ^2^, and *p* ≤ 0.05 to assess degree of impact on the outcome variable to inform the choice of variables to include in the multivariable logistic regression model [49]. To ensure sufficient events per independent variable in the multivariable models, the ratio was set at a maximum of 10 : 1 [49–51]. Effect sizes are presented as odds ratios (OR) with 95% CI and *p* values. Variables were considered significant if *p* < 0.05, but *p* values between 0.01 and 0.05 were interpreted with caution due to multiple comparisons. The McKelvey and Zavoina *R* ^2^ was used to examine explained variability in the multivariable models. Its calculations are based on predicting a continuous latent variable underlying the observed 0-1 outcomes of data but need to be interpreted with caution compared to the adjusted *R* ^2^ in the Ordinary Least Squares regression [52, 53].

### 2.7. Tests of Statistical Assumption

Basic assumptions for logistic regression must be tested and reported [29, 49, 54]. One assumption is linearity in the logit for any continuous independent variables [49, 51]. For this we performed a linktest [55, 56]. The independent variables were also investigated for multicollinearity by means of the tolerance value that indicates the variables' uniqueness in explaining variation, where zero means perfect collinearity between variables. Perfect collinearity makes it impossible to obtain a unique estimate of regression coefficients for the involved variables [49, 56]. A definite cut-off criteria for “too much” multicollinearity do not exist. However, it is suggested that a value below 0.1 is problematic [57]. Assessments of the overall model fit were conducted by using the Hosmer-Lemeshow test [49, 51, 58]. Another assumption is independence between the observations. Patient observations collected in NHs might be described as clustered data and thereby correlated [59]. Consequently, the multivariable logistic regression models were tested against a mixed effects logistic regression model with the NH units treated as a random effect to investigate whether this further improved the model. STATA and the xtlogit command provide a likelihood-ratio test for the null hypotheses that the NH unit-level variance is significantly different from zero. In addition, the mixed effects logistic regression model makes it possible to investigate variance on two levels, the level of the individual patient versus the level of the NH unit [29, 60]. The Akaike information criterion (AIC) and the Bayesian information criterion (BIC) were used to compare model fit of the multivariable models and the mixed-effect models [30].

No replacements were made for missing data; thus, the number of patients varies between the different analyses. Statistical analyses were performed using STATA version 13 (StataCorp LP, Texas, USA).

### 2.8. Ethical Considerations

The study was approved by the Regional Committee for Medical and Health Research Ethics (REK) (2013/1802/REK North) and by The Norwegian Social Science Data Services (36482/2/MB). An essential ethical consideration in this study was whether or not informed consent should be obtained from patients or their representatives. After evaluating the overall project, REK authorized RNs with dispensations from the duty of confidentiality to gather relevant patient health information (proxy data). Since dispensation was given, patient consent was not obtained. The study was performed in accordance with the Helsinki Declaration.

## 3. Results

### 3.1. Characteristics of the Patients

The study included all patients (*n* = 261) within eligibility criteria from 20 NH units. Patient characteristics are presented in Table 1.

### 3.2. Constipation

There were 61 (23.4%) patients with constipation.  Table 2 shows the result from the univariable logistic regression analyses. Because of the 10 : 1 ratio criteria, only six of the variables were included in the multivariable model. The variables with the highest impact (log likelihood and McFadden's *R* ^2^) on the dependent variable in the univariable analyses and/or variables considered as clinical significant were included in the multivariable logistic regression model (Table 2).

#### 3.2.1. Mixed Effect Logistic Regression: The Constipation Model

The results are presented in Table 2. The likelihood-ratio statistic for the constipation model was 1.97 giving *p* = 0.08. Thus, the variance between NH units did not have a significant influence on the results, and thereby a multilevel model was not required. The analyses resulted in an intracluster correlation coefficient (ICC) = 0.097, indicating 90.3% of the variance in the data being on the individual patient level. The analyses comparing the multivariable logistic model with the mixed effect logistic model resulted in AIC and BIC values for the logistic model of 198.47 and 222.29 and for the mixed model 198.50 and 225.72, respectively. Lower AIC and BIC values indicate the better fit. This means that the result indicates a slightly better fit for the multivariable logistic regression model compared to the mixed effects model [30]. Hence, below we will present adjusted ORs from the multivariable logistic regression model (Table 2).

#### 3.2.2. Adjusted Results

The results show that the odds of constipation increase with an OR of 1.69 for each unit increase on the Balance scale, and OR of 1.34 for each unit increase on the urinary incontinence scale. Patients who were diagnosed with hypothyroidism had a higher risk of constipation (OR 8.59) compared with patients not diagnosed with hypothyroidism. Being diagnosed with Parkinson's disease resulted in an OR of 7.03 compared to patients not diagnosed with Parkinson's disease. This final model resulted in a McKelvey and Zavoina's *R* ^2^ of 0.312. This means that 31.2% of the total variability of constipation among patients can be explained by the variables in the model.

### 3.3. Use of Laxatives

The use of laxatives is reported in Table 3. There were 175 (67.1%) patients using laxatives regularly as reported on the drug charts. Forty-six (75.4%) of the patients defined by the nurses as constipated used laxatives regularly. As shown in Table 3, we found a rather huge difference in the use of enemas as prescribed in the patient record compared to the use of enemas as reported by RNs.  Table 4 shows the result from the univariable logistic regression analyses. Again, the variables with the highest impact (log likelihood and McFadden's *R* ^2^) on the dependent variable in the univariable analyses and/or variables considered as clinically significant were included in the multivariable logistic regression model (Table 4).

#### 3.3.1. Mixed Effect Logistic Regression: The Laxative Use Model

The results are presented in Table 4. The likelihood-ratio statistic was 0.21 giving *p* = 0.325, indicating also here that the variance between NH units did not have a significant influence on the results, and thereby a multilevel model was not required. The analyses resulted in an ICC = 0.031, indicating 96.9% of the variance in the data being on the individual patient level. The analyses comparing the multivariable logistic model with the mixed effects logistic model resulted in AIC and BIC values for the logistic model of 292.62 and 356.15 and for the mixed model of 294.41 and 361.47, indicating best fit for the multivariable logistic regression model [30]. Hence, we will also here present adjusted ORs from the multivariable logistic regression model (Table 4).

#### 3.3.2. Adjusted Results

The results shows that OR for laxative use increases by 1.22 for each unit increase on the COMM scale, and with an OR of 1.23 for each increase in number of medications other than laxatives. Being engaged in activities between 1/3 and 2/3 of daytime resulted in a protective effect (OR = 0.28) compared to the patients not engaged in activities at all. Taking antidementia medications gave a protective effect with an OR = 0.17 compared to patients not taking antidementia medications. This final model resulted in a McKelvey and Zavoina's *R* ^2^ of 0.369, explaining 36.9% of the total variability.

### 3.4. Results of the Test for Statistical Assumptions

*Linearity in the Logit*. For both regression models, the linktest was not significant with *p* = 0.802 for the constipation model, and *p* = 0.245 for the laxative use model. This means that the model was properly specified and that the assumption of linearity was fulfilled [55, 56]. 

*Multicollinearity*. For the model with “constipation” as the dependent variable no adjustment of the model was made as a result of the tolerance test. The variable with the lowest value was “CHESS” with the value 0.89. For the model with “Laxative use” as the dependent variable, ADLlf had a tolerance value of 0.27, which is rather low but not surprising since ADLlf includes a range of measures that might interfere with the uniqueness of the variable in the multivariable analyses. However, after investigating the fit of different alternatives with and without ADLlf, and the variables “Type of food”, “Maximum walking distance”, and “Locomotion”, we chose to keep ADLlf in the model and exclude “Maximum walking distance”. This maneuver changed the tolerance value for ADLlf from 0.27 to 0.33. Either way, the models were stable considering *p* values and confidence intervals in the different alternatives. The result from the* Hosmer-Lemeshow test* on the final models resulted in a goodness-of-fit *χ* ^2^ = 5.38, *p* = 0.716, for the constipation model and goodness-of-fit *χ* ^2^ = 6.11, *p* = 0.635, for the laxative use model. This means that both models fit the data well [58].

## 4. Discussion 

### 4.1. Constipation

The prevalence of constipation was 23.4% among NH patients. Comparison of prevalence rates in general is difficult because the definitions of constipation vary. In the NH population, there is even larger variation amongst estimates of constipation, from 10% [11] up to 72% [6, 8]. In this study, there was no significant association between either age or gender and constipation among NH patients. This is different compared to the general population where constipation is more prevalent among women and where age is considered a risk factor [3, 4]. Since two reviews have identified the age of 65–70 years as when there is a particular increase in prevalence [4, 12], this study investigated age both as a continuous variable and as a categorical variable grouping patients on the bases of age with emphasis on age groups identified in the above-mentioned reviews. Either way, age was not significantly associated with constipation. This might mean that when living in a NH, factors other than age and gender are of importance.

Hypothyroidism and Parkinson's disease were significantly associated with constipation. This is consistent with findings in two reviews [4, 6]. These publications additionally identified stroke/cerebrovascular disease as a risk factor for constipation, which was not in this study. This might be explained by the rather small subgroup with these conditions and thereby a lack of power to explain associations between constipation and stroke. The same reviews [4, 6] identified reduced mobility and functional decline as risk factors for constipation. In our study, ADL lost its significance in the multivariable analyses. The rest of the variables available in interRAI LTCF measuring function and mobility were not significant in the univariable analyses or had too little impact on constipation to be considered for the multivariable analyses. On the other hand, the condition with the strongest impact on constipation was “balance.” Together, these findings suggest that balance problems are of greater importance than ADL deficiencies and immobility in the understanding of constipation.

Type of food (regular, soft/liquid), body mass index (BMI), and dehydration also had too little impact to be considered for the multivariable analyses. It is often suggested that insufficient diet, hydration, fiber, and physical activity are associated with constipation, but the evidence behind these factors is inconsistent and of low to medium quality [4, 6]. If this is the case, our results confirm these factors having a weak impact on constipation. However, Leung et al. [4] conclude that increasing fiber, exercise, and fluids might benefit patients with actual deficiencies. Our study also identified urinary incontinence as a risk factor for constipation. The association between urinary incontinence and constipation can be linked to common muscular and neurological processes regulating continence, defecation, and urinating. It might also be a result of an adverse effect from drugs used for urinary incontinence.

### 4.2. Use of Laxatives

In this study 67.1% of the patients used laxatives regularly. Other studies have reported regular use of laxatives in NHs from 55.3% to 83.6% [7, 24, 31, 61, 62]. Only number of drugs and ability to communicate remained significant risk factors in the adjusted analyses. The number of drugs as a risk factor for laxative use is found in several other studies [7, 24, 31]. Opiates were the only drug significantly associated with laxative use in the univariable analyses but lost significance in the adjusted analyses. These findings are opposite to the findings by Fosnes et al. [8] among NH patients in another part of Norway. They did not find a significant association between number of drugs and laxative use but found some antidepressants and benzodiazepine derivates as independent predictors. van Dijk et al. [22] found the overall adverse effect of drugs on constipation to be an overestimated risk.

As far as we know the association between laxative use and ability to communicate has not been reported before. This is an interesting result indicating that patients having problems making themselves understood, and understanding others, are more likely to use laxatives. The bowel is a sensitive organ that gives signals when the rectum is full. If the patient has lost the ability to understand and to communicate their bowel habits or need to defecate, it might lead to bowel problems and a prescription for laxatives.

Being involved in activities from ≥1/3 to 2/3 of daytime and antidementia medications were protective factors. Antidementia medications have diarrhea as a known adverse effect, which may lead to a lower risk for laxative use. The covariate “time involved in activities” expresses the patient's involvement in activities either alone or in a group when the patient is awake and not receiving treatment or care related to activities of daily living. Hence, the result supports the hypothesis that active living is protective against constipation and the need for laxatives. It might also be that the most active patients are able to manage their bowel independently in terms of responding to the need to defecate. However, it is worth mentioning that none of the other covariates involving physical activity or ADL functioning were significantly associated with laxative use in the adjusted analyses. Immobility in general [23, 24] and loss of functional status have been found to be a significant risk factor for laxative use in other studies [8, 62].

Stroke was significantly associated with laxative use in the univariable analyses, but not in the adjusted analyses. Parkinson's disease did not reach the significance level. Other studies show varying results concerning the association between Parkinson's disease and use of laxatives, where Chen et al. [62], did not find a significant association with either diseases, but both Hosia-Randell et al. [24] and Harari et al. [23] found an association between Parkinson's disease and use of laxatives. When investigating the relationship with stroke and Parkinson's disease it is possible that the nonsignificant findings are due to the small number with these conditions in the sample.

An important finding is the differences in the reported prescriptions for microenemas, small enemas, and oil enemas in the patient record compared to what was reported as used by the RNs. This indicates that RNs give patients these drugs without prescription from the GP, which support the hypotheses that in NHs RNs handle bowel problems independently [61], including the administration of laxatives.

### 4.3. Constipation and Laxative Use

When investigating and comparing variance on the NH unit level and the patient level, our results show that the significant variability in constipation and laxative use among patients is largely explained by difference in patients characteristics/health deficiencies, for example, number of drugs, different medical diagnosis, or ability to communicate. Although interRAI LTCF offers a large number of variables, the results show a rather low explained variability of 31.2% (constipation) and 36.9% (laxative use). Hence, other variables should be considered. One possible variable to discuss is the overall care routines in the NH setting. Even though this study identified most of the variance at the patient level, most of the patients are dependent on care staff to compensate for the deficiencies that make them at risk for constipation and laxative use.

Constipation and laxative use might be considered a result of standardized routines where the patients have not received an individualized assessment or treatment for their bowel needs. This interpretation may be supported by the positive association between constipation and urine incontinence where care related to elimination in general is determined by care routines and not the patients' individual needs, possibly leading to a worsening in ability to maintain function. In spite of increased recognition of the importance of the application of individualized treatment and care in NHs, NHs with few nursing resources dedicated to the care of older persons might be based on standardization [63, 64] and routine [65]. Several studies have identified care culture, with standardized routines as a problem for individualized bowel care [18, 31].

### 4.4. Strengths and Limitations

A major strength is the use of interRAI LTCF with standardized and validated measures for investigating prevalence and associations. A study investigating and comparing reliability in the different interRAI instruments in 12 countries found the majority of the items to exceed standard cut-offs for acceptable reliability [36]. However, the different scales have shown varying results for validity and reliability [36, 45, 46, 66, 67], with the CHESS scale and the DRS scale as the two with the most variable results [67–69]. Another strength is that we have considered the effect of clustering and tested whether a mixed effect logistic regression model made a significantly better fit for the data.

A limitation is that we did not use ROME III criteria [70] when defining constipation among patients. InterRAI LTCF only considers two aspects of constipation: no bowel movements for three days, or problems with hard stools. On the other hand, the ROME III definition of constipation is problematic in this population because (1) many of the patients are treated with laxatives and (2) patients are cognitively impaired and might have a problem since ROME III uses a combination of subjective symptoms to define constipation which can be hard to verbalize for a cognitively impaired person. Another limitation in our study is that it did not include variables measuring the patients' fiber or calorie intake.

The use of a proxy, where the RNs filled in the interRAI LTCF based on their knowledge about the patients' health condition, and not the patients themselves, might be considered a limitation. The reliability and validity of proxy data is found to be high for tasks of daily living and health conditions that are easily observed and relatively low for conditions that are private and less likely to be reported [71]. In the NH setting, most of the patients have cognitive impairment, which make it difficult to answer questions or fill in questionnaires. However, in order to get a representative sample of the NH population, we chose to design a study with the use of proxy data.

Other limitations are that the relatively small sample might have impeded the investigation of association of some conditions, for example, Parkinson's disease, stroke, and multiple sclerosis, which were found significant in other studies. This might threaten the external validity of the study concerning the conditions in question. Only patients that according to their patient record used laxatives or other drugs regularly were defined as users in the analyses. Patients with an “on demand” prescription were defined as nonusers. Hence, patients defined as nonusers may have used laxatives or other drugs and thereby influenced the results.

## 5. Conclusion

The prevalence of constipation was 24.1%, and was associated with impaired balance, urinary incontinence, Parkinson's disease, and hypothyroidism. About 67% of the patients used laxatives regularly. Laxative use was associated with impaired ability to communicate and number of other drugs used. Antidementia drugs and being involved in activities were protective factors. Mixed-effects analyses of both the constipation model and the laxative use model identified variance between NH units as nonsignificant in explaining the total variance. Hence, variance in constipation and laxative use are mainly explained by different individual patient characteristics/health deficiencies. NH patients are dependent on care staff to compensate for health deficiencies. NHs with few nursing resources might perform care based on standardization and routines. Hence, standardized care might be an important factor in order to explain constipation and laxative use among patients.

## Figures and Tables

**Table 1:** Patients characteristics^1^, *n* = 261.

Age, years	84.7 (8.3)
Gender, female	173 (66.3)
CPS^2^ ≥ 2	177 (69)
BMI^2^	23.1 (5.1)
ADL^2^ long form	12.6 (9.3)
Locomotion	
(i) Walks without aid	52 (20.0)
(ii) Walks with aid (e.g., cane, crutches, rollator)	140 (53.8)
(iii) Wheelchair	56 (21.5)
(iv) Bed-ridden	12 (4.6)
Length of stay, years	2.3 (2.5)
Number of medical diagnoses	2.6 (1.5)
Number of drugs	7.0 (3.5)

^1^The results are given as mean (standard deviation (SD)) and number (proportion (%)).

^2^CPS = Cognitive Performance Scale, BMI = body mass index, ADL = activities of daily living.

**Table 2:** Associations between constipation and clinical health problems, medical diagnoses, and medications.

Variables	Univariable logistic regression^1^ OR (95% CI)^4^	*p* value	Multivariable logistic regression^2^ OR (95% CI)	*p* value	Mixed effects logistic regression^3^ OR (95% CI)	*p* value
Age (years)	1.00 (0.97–1.04)	0.906				
Gender (female)	1.06 (0.58–1.96)	0.842				
BMI	0.95 (0.89–1.01)	0.112				
Length of stay, years	1.07 (0.96–1.19)	0.255				
CPS^5^ (scale 0–6)	1.16 (0.99–1.37)	0.069				
Balance (scale 0–4)	1.69 (1.33–2.13)	<0.001	1.68 (1.25–2.27)	0.001	1.69 (1.23–2.32)	0.001
Urinary incontinence (scale 0–4)	1.44 (1.18–1.75)	<0.001	1.35 (1.06–1.71)	0.015	1.38 (1.07–1.78)	0.013
ADL^5^ long form (scale 0–28)	1.06 (1.03–1.09)	<0.001				
CHESS^5^ (scale 0–5)	1.41 (1.10–1.81)	0.007	1.23 (0.90–1.68)	0.187	1.29 (0.92–1.81)	0.142
Maximum walking distance						
0 = <5 m	0.44 (0.24–0.78)	0.006				
1 = ≥5 m						
Bed rails	2.54 (1.41–4.57)	0.002				
COMM^5^ (scale 0–8)	1.17 (1.04–1.31)	0.007				
Sleep during daytime	2.28 (1.21–4.30)	0.011				
Fatigue (scale 0–4)	1.37 (1.05–1.79)	0.021				
Time involved in activities						
No time	Reference					
<1/3 of daytime	0.69 (0.30–1.60)	0.392				
≥1/3 to 2/3 of daytime	0.64 (0.27–1.50)	0.306				
>2/3 of daytime	0.22 (0.06–0.76)	0.016				
Dehydrated	2.11 (1.10–4.05)	0.025				
Type of food						
0 = regular	2.27 (1.16–4.44)	0.017				
1 = soft/liquid						
Pressure ulcer	2.17 (1.08–4.36)	0.029				
Hypothyroidism	4.29 (1.26–14.59)	0.029	7.63 (1.86–31.34)	0.005	8.59 (1.79–41.21)	0.007
Fecal incontinence	1.87 (1.05–3.33)	0.034				
Parkinson's disease	3.03 (0.98–9.49)	0.055	5.96 (1.36–26.18)	0.018	7.03 (1.49–33.30)	0.014
Stroke	1.87 (0.89–3.77)	0.098	2.34 (0.94–5.80)	0.073	2.14 (0.79–5.74)	0.133

^1^Univariable logistic regression performed with constipation as dependent variable and explored by a range of covariates.

^2^Multiple logistic regression, covariates selected by direct variable selection with *p* ≤ 0.05 and/or the highest impact (Log likelihood, McFadden's *R* ^2^) on constipation in the univariable logistic regression analyses.

^3^Mixed effect logistic regression, NH units defined as grouping variable (cluster) to investigate the impact of NH units, and whether the grouping of data significantly affected the results. The estimated intracluster correlation coefficient, ICC = 0.097. The likelihood ratio test for testing if the data required a multilevel model resulted in *p* = 0.08 meaning that a multilevel model will not significantly improve the analyses of the data.

^4^Results are presented as odds ratios (OR), 95% confidence intervals (CI), and *p* values.

^5^CPS = Cognitive Performance Scale, ADL = activity of daily living, CHESS = Changes in Health, End-Stage Disease, Signs, and Symptom Scale, COMM = Communication Scale.

**Table 3:** Use of laxatives among patients, *n* = 261.

Laxative type	Patients using laxatives, *n* (%)
Use of laxatives as prescribed in the patients record	
Laxatives regularly^1^ and on demand^1^	187 (71.7)
Laxatives regularly only	175 (67.1)
(i) Stimulant laxative (A06A B)^2^	87 (33.3)
(ii) Osmotic laxatives (A06A D)	143 (54.8)
(iii) Softening laxatives (A06A A)	1 (0.4)
(iv) Microenema (A06AG02 or A06AG11)	4 (1.5)
(v) Bulk laxatives (A06A C)	0 (0)
(vi) Oil enema (A06AG04)	0 (0)
(vii) Minienema (A06AG10)	0 (0)
Use of enemas as reported by nurses	
(i) Microenema (A06AG02 or A06AG11)	78 (30.0)
(ii) Oil enema (A06AG04)	10 (3.9)
(iii) Minienema (A06AG10)	6 (2.3)
Number of laxatives per patient^3^	
0	76 (29.1)
1	88 (33.7)
2	58 (22.2)
3	35 (13.4)
4	4 (1.5)

^1^Regular use and on demand as prescribed in the patient record.

^2^Laxatives are grouped according to the Anatomical-Therapeutic-Chemical Classification System (ATC).

^3^Reported as regular use in the patient record together with the use of microenemas, oil enemas, and minienemas as reported by nurses.

**Table 4:** Associations between laxative use and clinical health problems, medical diagnoses, and medications.

Variables	Univariable logistic regression^1^ OR (95% CI)^4^	*p* value	Multivariable logistic regression OR^2^ (95% CI)	*p* value	Mixed effects logistic regression^3^ OR (95% CI)	*p* value
Age (years)	0.99 (0.96–1.03)	0.731	1.01 (0.97–1.05)	0.529	1.01 (0.97–1.05)	0.541
Gender (female)	1.36 (0.79–2.33)	0.265	1.42 (0.74–2.73)	0.288	1.38 (0.70–2.72)	0.346
BMI^5^	1.02 (0.97–1.08)	0.443				
Length of stay (years)	1.14 (1.00–1.29)	0.046	1.04 (0.89–1.22)	0.579	1.05 (0.89–1.23)	0.573
CPS (scale 0–6)	1.13 (0.98–1.32)	0.101				
Locomotion						
0 = walking with/without help	3.25 (1.60–6.60)	0.001	1.74 (0.65–4.68)	0.269	1.61 (0.56–4.65)	0.378
1 = wheelchair/bedridden						
Urinary incontinence (scale 0–4)	1.21 (1.03–1.41)	0.020	1.06 (0.81–1.39)	0.646	1.05 (0.80–1.39)	0.711
ADL^5^ long form (scale 0–28)	1.07 (1.03–1.10)	<0.001	1.03 (0.97–1.09)	0.325	1.03 (0.97–1.09)	0.310
Time involved in activities						
No time	Reference		Reference		Reference	
<1/3 of daytime	0.31 (0.11–0.88)	0.028	0.39 (0.12–1.26)	0.117	0.39 (0.12–1.29)	0.122
≥1/3 to 2/3 of daytime	0.23 (0.08–0.33)	0.006	0.28 (0.08–0.93)	0.037	0.28 (0.08–0.95)	0.042
>2/3 of daytime	0.25 (0.08–0.79)	0.017	0.38 (0.10–1.46)	0.159	0.36 (0.09–1.47)	0.155
Opiates	3.62 (1.69–7.76)	0.001	1.33 (0.55–3.24)	0.529	1.42 (0.55–3.66)	0.472
Antidementia drugs	0.22 (0.07–0.68)	0.008	0.17 (0.05–0.66)	0.010	0.17 (0.04–0.68)	0.012
Number of drugs	1.17 (1.07–1.28)	0.000	1.23 (1.09–1.39)	0.001	1.24 (1.09–1.41)	0.001
COMM^5^ (Scale 0–8)	1.17 (1.04–1.31)	0.008	1.22 (1.03–1.45)	0.023	1.23 (1.03–1.48)	0.025
Stroke	2.77 (1.17–6.53)	0.020	2.00 (0.74–5.38)	0.170	2.10 (0.75–5.88)	0.158
Parkinson's disease	6.22 (0.79–48.67)	0.082	8.32 (0.72–95.76)	0.089	8.29 (0.69–99.58)	0.095
Type of food						
0 = regular	2.18 (1.03–4.61)	0.042	0.72 (0.28–1.85)	0.496	0.75 (0.28–1.97)	0.555
1 = soft/liquid						
Fecal incontinence	1.69 (0.99–2.90)	0.054	0.55 (0.22–1.35)	0.191	0.56 (0.22–1.41)	0.219

^1^Univariable logistic regression performed with laxative use as dependent variable and explored by a range of covariates.

^2^Multiple logistic regression, covariates selected by direct variable selection with *p* ≤ 0.05 and/or the highest impact (Log likelihood, McFadden's *R* ^2^) on laxative use in the univariable logistic regression analyses.

^3^Mixed effect logistic regression, NH units defined as grouping variable (cluster) to investigate the impact of NH units, and whether the grouping of data significantly affected the results. The estimated intracluster correlation coefficient, ICC = 0.031. The likelihood ratio test for testing if the data required a multilevel model resulted in *p* = 0.325 meaning that a multilevel model will not significantly improve the analyses of the data.

^4^Results are presented as odds ratios (OR), 95% confidence intervals (CI), and *p* values.

^5^CPS = Cognitive Performance Scale, ADL = activity of daily living, COMM = Communication Scale.
